# The floating duck syndrome: biased social learning leads to effort–reward imbalances

**DOI:** 10.1017/ehs.2024.20

**Published:** 2024-04-29

**Authors:** Erol Akçay, Ryotaro Ohashi

**Affiliations:** Department of Biology, University of Pennsylvania, Philadelphia, PA, 19104, USA

**Keywords:** Social learning, effort–reward imbalance, overcommitment, cultural evolution

## Abstract

An increasingly common phenomenon in modern work and school settings is individuals taking on too many tasks and spending effort without commensurate rewards. Such an imbalance of efforts and rewards leads to myriad negative consequences, such as burnout, anxiety and disease. Here, we develop a model to explain how such effort–reward imbalances can come about as a result of biased social learning dynamics. Our model is based on a phenomenon that on some US college campuses is called ‘the floating duck syndrome’. This phrase refers to the social pressure on individuals to advertise their successes but hide the struggles and the effort put in to achieve them. We show that a bias against revealing the true effort results in social learning dynamics that lead others to underestimate the difficulty of the world. This in turn leads individuals to both invest too much total effort and spread this effort over too many activities, reducing the success rate from each activity and creating effort–reward imbalances. We also consider potential ways to counteract the floating duck effect: we find that solutions other than addressing the root cause, biased observation of effort, are unlikely to work.

## Graphical abstract

**Figure:**
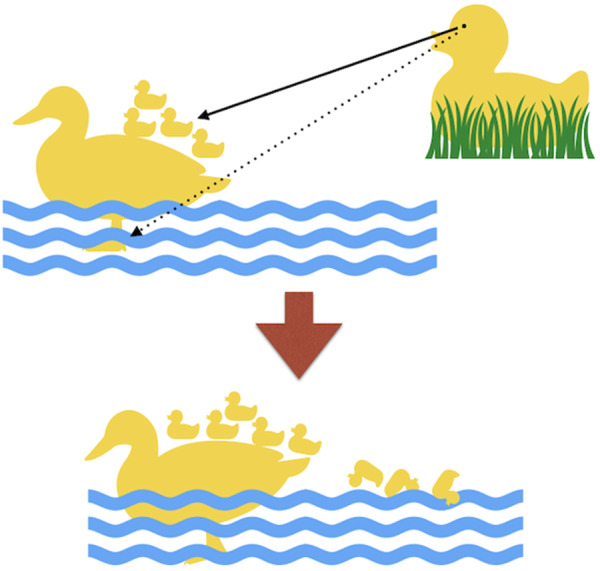

## 1. Introduction

Modern life constantly calls upon us to decide how to divide our time and energy between different domains of life, including school, work, family and leisure. How we allocate our time and energy between the domains, how many different activities we pursue in each domain, and what the resulting rewards are, have profound effects on our mental and physical health. For example, occupational health research shows that persistent mismatches between effort at work and the material and social rewards from it can cause mental and physical health problems (Siegrist, 1996; Nordentoft et al., 2020). A common component of such a mismatch is overcommitment, or taking on too many tasks, which causes individuals to overextend themselves and underachieve rewards. Overcommitment in work and school settings is well documented and is frequently associated with adverse outcomes such as depression and anxiety (Wege et al., 2017; Adam, 2021; Porru et al., 2021). Overcommitment and the resulting pressure to multitask also contributes to incentives for academic dishonesty (Lavy, 2023). In all, overcommitment and effort–reward imbalances contribute significantly to the heavy toll of mental health problems on college campuses that claim more than 1000 students’ lives every year (Mistler et al., 2012).

A related phenomenon is known to students as the floating duck syndrome on some US campuses. This term, originally coined at Stanford University, compares a student's career with the seemingly effortless glide of a duck on the surface of the water while paddling furiously underwater, desperately getting by (Stanford University Student Affairs, 2022). It is meant to capture the twin pressures to succeed while making it seem easy. Other comparable terms exist in other schools (e.g. ‘Penn Face’ at the University of Pennsylvania, or the more descriptive ‘effortless perfection’ at Duke and Princeton Universities, Scelfo, 2015). The result of these pressures is that students often observe their peers’ successes but less often see the effort that was put in or the failures experienced. This phenomenon is often exacerbated by social media platforms and institutional public relations, which make successes more visible but not necessarily failures or the effort spent to achieve successes. This creates a visibility bias, which refers to a situation where certain types of information or actions are more readily observable than others, creating a skewed perception of others’ behaviors. In environments where individuals learn from others, such visibility biases can skew the information available to individuals. This can cause systematic distortions of behavior even in populations of agents that behave optimally given their information (Han et al., 2023; Hirshleifer & Plotkin, 2021; Akçay & Hirshleifer, 2021).

In this paper, we propose that the bias against the visibility of effort, i.e. the paddling of the ‘floating duck’, is a mechanism that causes overcommitment and an effort–reward imbalance amongst students. We develop a mathematical model of social learning in the presence of visibility biases. Specifically, we model a world where individuals try to make optimal decisions about their work effort, but have incomplete information about the difficulty of world, that is, how much effort it takes to succeed in a given activity. They therefore try to infer this property by observing the successes and the effort levels of their peers. This inference is complicated by the further assumption that each individual's costs from effort (or utility from success) is private information. We show that under these conditions, the pressure to appear to be succeeding effortlessly leads others to underestimate how difficult the world is. Somewhat paradoxically, this causes individuals to invest too much total effort, while at the same time dividing it between too many different activities. We show that this can indeed lead to higher numbers of successes, but at the cost of reducing overall utility and a mismatch between expected and realised rewards.

The contribution of our paper is to provide an account of the floating duck syndrome and its negative consequences based on a rational actor model. Most existing accounts of this phenomenon rely on the idea of social comparison and negative emotions like envy (Festinger, 1954; Dijkstra et al., 2008; Verduyn et al., 2020). Instead, our model considers agents as making optimal decision given their information. In this account the negative consequences of the floating duck syndrome follow not from emotional processes, but from the fact that in the presence of biased information, individuals’ subjectively (given their beliefs) optimal decisions will not be optimal in an objective sense. As a result of the biased information, individuals in our model mistakenly expect more reward for their effort which is not realised, leading to effort–reward imbalances. This provides a new hypothesis to understand the root cause of overcommitment and burnout on campuses and suggests new points of intervention to address the problem by helping individuals make better decisions about how to invest their effort. More broadly, our paper contributes to the growing literature that regards educational outcomes as resulting from individuals responding to perceived returns from their effort. Our own experiences as student and faculty, as well as qualitative evidence (McClelland & Case, 2023) suggest that students are aware of tradeoffs and opportunity costs they face in allocating effort to their studies and other activities, and try to make optimal choices. Recent studies have started to quantify returns to study effort in terms of academic achievement (Stinebrickner & Stinebrickner, 2008; Ersoy, 2021). These studies show that individuals’ beliefs about these returns are not always accurate (e.g. metaanalysis in Pinquart & Ebeling, 2020) and experimentally changing these beliefs can result in changes in effort investment and outcomes (Ersoy, 2023; Wright & Arora, 2022; Rury & Carrell, 2023). Our model adds to this literature by placing it in the context of social learning from peers. We show that social learning causes autonomous dynamics in beliefs that can converge to persistently inaccurate beliefs if there are visibility biases. Optimally behaving agents subject to such inaccurate beliefs will then experience systematically distorted and suboptimal outcomes. We also evaluate some potential solutions to this problem and show that solutions that do not address the root cause, biased social learning, are unlikely to succeed.

## 2. Basic model of optimal investments

We consider a simple model where an individual has to decide how much total effort to invest into activities, and how many activities to attempt. An activity can be a course, a student club or a job. To keep the model simple, we assume each activity is independent from others, and the individual succeeds or fails in each activity only as a function of the effort put into that activity. We assume that success in each activity yields a unit reward, which can be getting a good grade, getting an award or simply a sense of accomplishment. Effort, in turn, can mean both tangible quantities such as time investment, but also less tangible quantities such as energy and enthusiasm. What is important is that effort invested into activities has a cost, either material costs (e.g. the financial costs of activities) or opportunity costs (e.g. not investing enough time in leisure).

We denote the total (aggregate) effort level as *X*_*A*_. We assume each individual attempts *n* activities, and for simplicity assume that the total effort gets distributed evenly across activities, so each activity receives effort
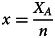
or equivalently the number of activities attempted is
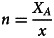
For mathematical convenience, we take *n* to be a continuous variable in our main analysis. Success in each activity is both a function of the effort (*x*) invested into that activity and the ‘difficulty’ of the world, which we denote by *θ*; specifically, we denote success probability with a twice differentiable function *f*(*x*, *θ*), with
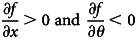
These assumptions mean that investing more into an activity increases the chance of success in that activity for a given difficulty *θ*, while a more difficult world (higher *θ*) reduces the chance of success for a given investment of effort into an activity. Again for simplicity, we assume that all activities have the same difficulty *θ* and the same payoff from success which we set to 1. The total expected number of successes, *s*(*X*_*A*_, *x*, *θ*) for given total and per activity effort levels (*X*_*A*_ and *x*, respectively) is then given by:1
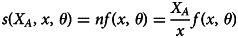


Finally, we assume that the effort comes at an cost *c*(*X*_*A*_, *k*), a twice differentiable function with
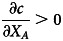
so the cost is increasing in total effort. We assume the cost function varies between individuals: different individuals might experience different levels of cost from a given effort (or equivalently, different utility from success in activities). This is accounted for by the second argument, *k*, in the cost function. We assume this cost parameter *k* is privately known to each individual, and not directly observable by others.

**Figure 1.**
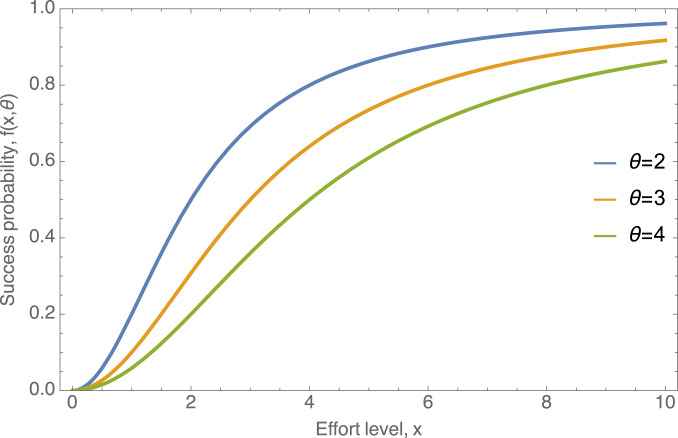
An example for the success function 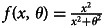
 plotted for different values of *θ*, illustrating that the success function is increasing with effort *x*, but decreasing with the difficulty of the world, *θ*. Because this function gives the probability of success, it must be bounded between 0 and 1 (more generally, the utility from a single activity must be bounded), which gives it a characteristic sigmoidal shape.

Although many of our results apply generally for suitable functional forms of *f*( ⋅ ) and *c*( ⋅ ), for ease of exposition we will work in part with a convenient set of functions. In particular, we will use the Tullock contest function (Tullock, 1980) for the success probability *f* and a quadratic cost function *c*:2
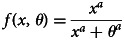
3
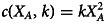
where *a* (assumed to be greater than 1) is a shape parameter. This success function can be interpreted as each activity being a ‘contest against the world’ where the ‘world’ invests *θ* effort; increasing this *θ* lowers success probability. This function is plotted in Figure 1 for different values of *θ* and for the shape parameter *a* = 2. Higher values of *a* make the success function more threshold-like (switching from 0 to 1 more rapidly around the difficulty level).

### 2.1. Optimal efforts under perfect information

We assume that each individual chooses *X*_*A*_ and *x* to maximise their total utility given by *s*(*X*_*A*_, *x*, *θ*) − *c*(*X*_*A*_, *k*). Suppose that the individual knows the difficulty of the world *θ*. (We assume every individual knows their own *k*.) Then, choosing *X*_*A*_ and *x* presents no problems: the optimal allocations must satisfy the following first order conditions:4
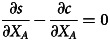
5
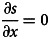


Solving these first-order conditions gives us optimal total and per activity effort which we will denote by 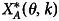 and 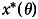, respectively. Note that the per activity allocation 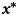 does not depend on the cost parameter *k*, since the cost is only a function of the total effort, not how many activities it is allocated to. These optimal effort levels are functions of *θ* because the success function *f*(*x*, *θ*) depends on *θ* and so does its derivatives. Substituting the success function *s*(*X*_*A*_, *x*, *θ*) into the equations (4) and (5), we can write:6
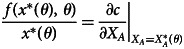
7
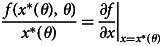


We also need to check that the second-order conditions
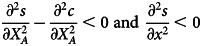
are satisfied to ensure that the solution to the first-order conditions are a maximum and not a minimum of the individual utility. The former implies that we need
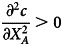
since
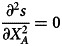
In other words, the cost of total effort has to be accelerating. Likewise, the other second order condition implies
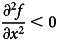
In other words, the marginal increase in the success probability in an activity must be declining with the effort put into that activity.

When the success function is given by the Tullock contest function (equation (2)) and the cost function is quadratic, the optimal total and per activity effort levels are given by:8
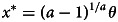
9
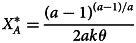


**Figure 2.**
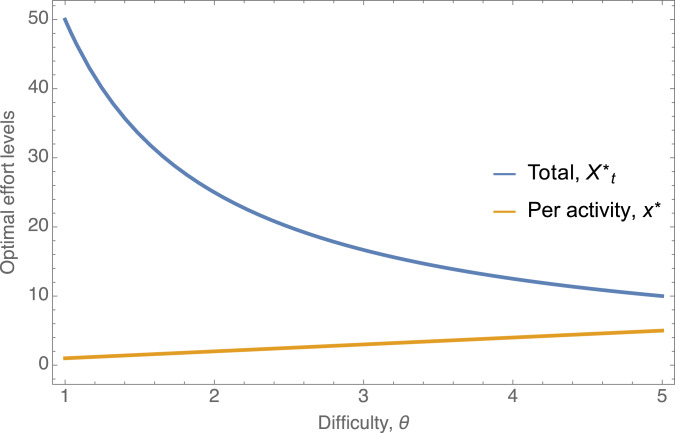
Optimal total (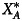) and per activity (x*) efforts as a function of *θ*, calculated using the success function depicted in Figure 1, 
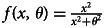
 and a cost function 
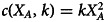
, with *k* = 1/200.

These solutions, depicted in Figure 2, show that the per activity effort increases with difficulty *θ* while the total effort decreases. The fact that the overall effort is declining in the difficulty of the world, *θ*, might seem counterintuitive but it stems directly from the fact that difficulty level only affects the rewards from effort, not the cost. Making the world more difficult reduces the rewards from investing more effort, and therefore individuals have a reduced willingness to invest. In the Supplementary Information (SI) section SI.1, we show that these results hold more generally than these particular functional forms, specifically they hold for any kind of sigmoidal success function. We also show that the number of successes is always monotonically decreasing in the difficulty of the world.

### 2.2. Inferring the difficulty of the world in a heterogeneous population

Now we consider a world where individuals know the general shape of the success function *f*(*x*, *θ*) but do not know how difficult the world is, i.e. the true value of *θ*, which we will call *θ*_*r*_ to distinguish from its inferred value. In order to make optimal effort investing decisions, individuals need to infer the value of *θ*. We now show that they can so do if they observe both the successes and overall effort levels.

From inequality (SI.5), we know that the expected number of successes 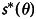 is monotonically decreasing with *θ* for a given cost parameter *k*. This means that if one observed the actual number of successes (or a representative sample) of individuals with known cost parameter *k*, one could mathematically invert the function 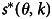 to obtain an unbiased estimate of the difficulty of the world, which we will term *θ*_*est*_(*s*_*obs*_), where *s*_*obs*_ is the observed number of successes.

However, because individual cost parameters *k* are idiosyncratic and privately known, an individual needs more information than just the observed number of successes, *s*_*obs*_. Intuitively, this is because the same number of successes can be obtained either by someone who has low effort costs and invests a lot of total effort in a difficult world, or by someone with high effort costs and invests relatively little in an easy world. Therefore, individuals also observe need to observe the total effort of others, which we denote by *X*_*A*,*obs*_. We can then rewrite the first order condition (7) as:10
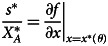
Note that the right-hand side of the above equation depends on *θ* but not on *k*. This means that if an individual knows the true difficulty level of the world and is behaving optimally, the ratio of their expected successes to their optimal total effort can be used to infer the true difficulty level *θ*_*est*_. Specifically, if one observes *s*_*obs*_ successes and *X*_*A*,*obs*_ total effort invested, one can solve the following equation to infer the estimated difficulty of the world, *θ*_*est*_:11
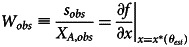
where we defined *W*_*obs*_ as the observed ratio of successes to total effort. We will call this quantity the observed success rate per unit overall effort. Equation (11) can give an unbiased estimate of *θ*, provided the observed quantities on the left-hand side are also unbiased. In particular, using the Tullock contest function (equation (2)) for the success function, we can write the estimated *θ*_*est*_ as:12
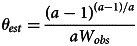
If the success rate *W*_*obs*_ is observed without bias, i.e. equal to the ratio obtained from solving for the first-order condition (eq. (10)), and the observed individuals know the true value *θ*_*r*_, one can show that:13
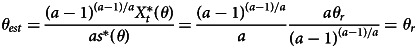
In other words, in a world with unbiased observation of accurately informed individuals’ successes and total efforts, it is possible to accurately infer the true difficulty of the world. In the next section, we will show a stronger result, namely that even if the population is initially misinformed (have an inaccurate estimate of *θ*), unbiased social learning dynamics will over generations converge to the true value.

## 3. Social learning dynamics with visibility bias

Next we consider a simple model of a social learning dynamic and show that it can converge on inaccurate beliefs when individuals hide or under-report their true efforts, or effort is otherwise hard to observe fully. This is the essential element of the floating duck phenomenon: one does not see the furious paddling underwater. This is a kind of visibility bias (Han et al., 2023), which is related to but is distinct from tranmission biases considered in social learning literature which mostly deal with non-random selection of models to learn from (Kendal et al., 2018).

Our dynamic proceeds as follows: at each time step *t*, a naive individual enters the world. They know their own cost function, including their cost parameter, *k*, and the functional form of the success function *f*(*x*, *θ*), but do not know the value of *θ*_*r*_, the (constant) true difficulty of the world. To infer the difficulty of the world, the naive individual observes the individual from the previous generation (time *t* − 1), specifically their success rate *W*_*obs*_. They do not observe the previous generation individual's cost parameter *k* (nor need to, given *W*_*obs*_, to infer *θ*_*est*_). Note that in this setting, the expected success rate is a function of both the true difficulty level of the world, *θ*_*r*_, and the estimated one, *θ*_*est*_. The latter is what individuals use to solve their optimality conditions, while the former determines the actual success probability given the efforts invested.

To model the floating duck phenomenon, we assume that individuals systematically under-report their effort level by a constant factor of *δ* (0 ≤ *δ* < 1). In other words, we assume:14
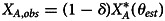
where 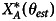 is the optimal total investment when the difficulty of the world is estimated as *θ*_*est*_. This in turn means that the observed success rates *W*_*obs*_ are inflated from their true values by a factor
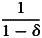
When the success and cost functions are given by the functions in (2) and (3), this means that the expected observed success rate, when generation *t* − 1 estimates the difficulty of the world as *θ*_*est*_(*t* − 1) while it is in reality *θ*_*r*_, is given by:
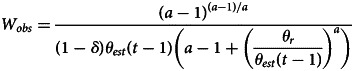


Substituting this into equation (11), we obtain for the estimate of the difficulty in generation *t*, *θ*(*t*):15
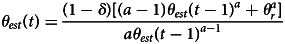


This equation allow us to calculate what the current generation's estimate *θ*_*e*_*st*(*t*) will be given the previous generation's estimate. This gives us a discrete time dynamical system, or equivalently, a one-dimensional discrete map (Strogatz, 1994). For such a discrete time dynamic, one of the main quantities of interest is its fixed points or equilibria, which are points where the dynamics do not lead to any change from one generation to the next. Further, a fixed point or equilibrium can be stable or unstable, meaning that the dynamics might return to the fixed point when perturbed away from it, or not. Stable fixed points can be expected to be the outcomes in the long run of discrete time dynamics such as the one given in (15).

**Figure 3.**
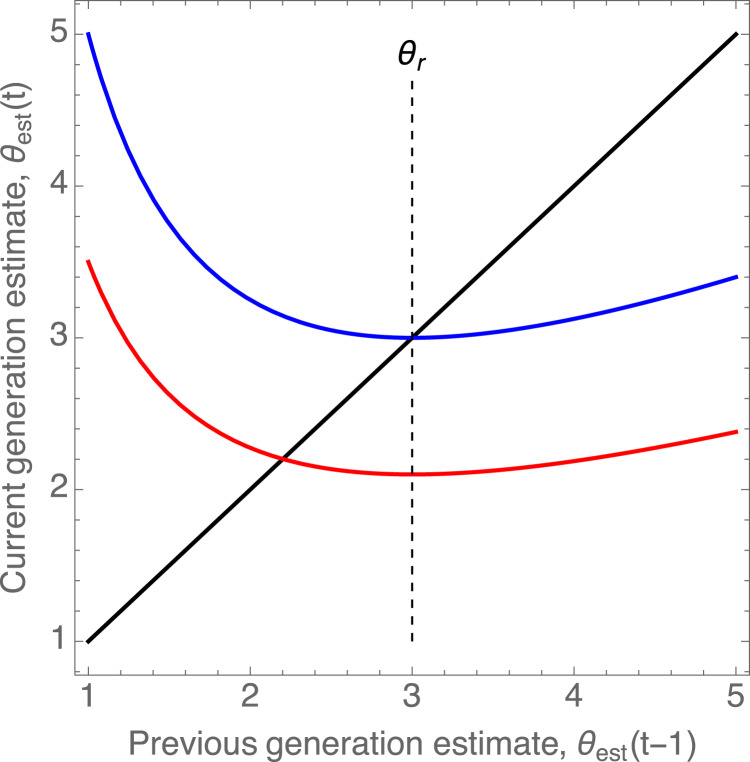
Graphical depiction of the one-dimensional map in equation (15) that describes the social learning dynamics, with *α* = 2 and *θ*_*r*_ = 3. The blue and red curves show the current generation's estimate *θ*_est_(*t*) given the previous generation's, *θ*_est_(*t* − 1), for *δ* = 0 (no under-reporting) and *δ* = 0.3 (30% of effort gets unreported), respectively. The point at which each curve intersects the 45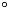 diagonal is an equilibrium of this dynamic. The blue curve intersects the diagonal at exactly *θ*_*r*_ (marked with a vertical dashed line), meaning that without under-reporting, the social learning dynamics converge to the true value of the difficulty of the world. In contrast, the red curve's intersection with the diagonal lies below *θ*_*r*_, indicating that under-reporting causes the equilibrium estimate of the difficulty to be lower than the true difficulty.

The fixed point of the map in estimated *θ*_*e*_*st* can be found by setting in equation (15) 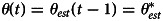 and solving for 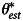, which yields:16
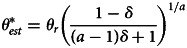
For *δ* > 0, i.e. with under-reporting of effort, and *a* > 1 the factor multiplying *θ*_*r*_ on the right-hand side is going to be less than one, meaning that the equilibrium estimate 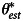 from the social learning dynamics is going to be less than the true difficulty of the world, *θ*_*r*_ (Figure 3). In other words, the floating duck syndrome will make individuals underestimate the difficulty of the world in the long run.This fixed point is stable when the derivative of the right-hand side of equation (15) with respect to *θ*_*est*_(*t* − 1), evaluated at the fixed point, is between −1 and 1, which yields:17
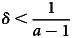

If this condition is satisfied, the learning dynamics converge to a stable value. If it is not satisfied, the dynamics converge to a stable cycle, as is common in discrete maps. Note that the parameter *a* determines how steep the Tullock contest function is (i.e. how quickly it switches from low success probability to high success probability). Therefore, this condition means that steeper success functions impose a stronger limit on under-reporting in order for learning dynamic to converge to a stable equilibrium.

**Figure 4.**
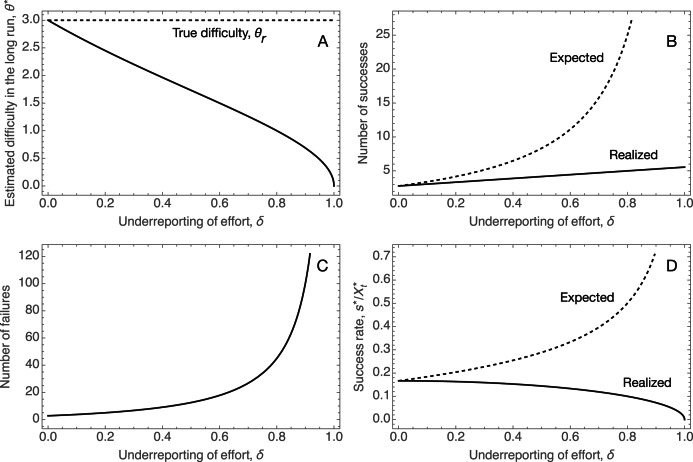
Illustration of the effects of the floating duck syndrome, using the Tullock contest function and quadratic cost functions (Eqns (2) and (3)) with *a* = 2, *k* = 1/200, and true difficulty of the world *θ*_*r*_ = 3, indicated with the dashed line in the first panel. Panels show the variation with under-reporting *δ* in: (a) the long-run estimate of the difficulty of the world, θ*m given the level of under-reporting (equation (16)); (b) the number of successes expected (dashed curve) and realised (solid curve) at optimal effort investment given the long-run estimate θ*; (c) the number of failures at this long-run estimate; and (d) the expected (dashed curve) and realised success rate at this long-run estimate. Panel (a) shows that as under-reporting of effort increases, the social learning dynamics in the long run underestimate the true difficulty of the world (depicted by the dashed line) more severely. This leads individuals to put in more total effort and spread this out over more activities (as shown in Figure 2). As a result, the mean number of successes increases (panel b), but so does the discrepancy between the number of successes individuals come to expect given the inferred estimate θ* (dashed curve in panel b) and the actual number of successes realised (solid curve in panel b). Likewise, the number of failures (activities attempted by did not succeed also increases with under-reporting (panel c). Because total effort increases faster than the realised number of successes, the realised success rate per effort decreases (solid surve in panel d) with under-reporting, despite the fact that individuals’ long-run estimates θ* make them expect higher success rates (dashed curve in panel d). This is an indication of growing effort–reward imbalance as the level of under-reporting increases.

Figure 4 plots against the under-reporting bias, *δ*, the fixed point estimate 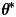 from the social learning dynamics, the mean number of successes and failures, and the mean success rate (i.e. the number of successes per total effort) when individuals estimate 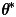 but the real difficulty is *θ*_*r*_. It shows several features: first, as indicated above, the fixed point of the social learning dynamics decreases as the under-reporting bias increases. Remarkably, this leads to an increased expected number of successes, despite the fact that the equilibrium effort per activity decreases as shown in Figure 2 for *α* = 2.^1^ This is because the total effort increases with the reduced inferred difficulty of the world, so while each activity is less likely to succeed, individuals try out many more activities. This is indicated by the fact that the mean number of failures (i.e. activities that were attempted but did not yield success) is sharply increasing with *δ*. Finally, while both the mean number of successes and total effort increase with *δ*, the success rate (their ratio) is decreasing as total effort gets diluted into more activities, many of which fail.These results show how the floating duck syndrome can lead to effort–reward imbalance (Siegrist, 1996), where individuals increase their effort relative to what would be optimal but do not achieve commensurate rewards, and indeed have to face the prospect of many more failures because the effort is diluted across too many activities. It also shows that this can happen while the absolute number of successes is going up, so focusing only on successes would not pick up this outcome. We believe that these results capture the essence of what is going on in many college campuses.

### 3.1. Short-term fixes do not solve the problem

Above we have shown that under-reporting of effort in a social learning dynamic causes individuals to underestimate the difficulty of the world, and leads to effort–reward imbalances It is tempting and to some degree inevitable to react to the adverse consequences of this underestimation by trying to lower the true difficulty so that it matches the expected difficulty inferred by the social learner. For example, it is tempting for faculty in a course to make assignments easier or give higher grades for the same performance. While this might resolve the short-term effort–reward imbalance, our model suggests that it will not work in the long term.

Specifically, we can model such a short term ‘fix’ by assuming that some outside agent (e.g. faculty or parents) anticipates the underestimation of *θ* and adjusts the difficulty of the tasks to lower the actual difficulty, *θ*_*r*_, so that it matches the estimated difficulty *θ*_*est*_ from social learning. Going through the same steps as in the previous section, we can calculate the expected success rate and the equivalent of the discrete map in equation (15), which in this case simplifies to:18
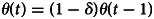


This means that even if an outside agent intervenes and lowers the true difficulty of the world to match the (initially) underestimated difficulty, agents in the next round that observe the (inflated) success rate from this artificially lowered difficulty setting will estimate an even lower difficulty level. The only equilibrium of that process is when *θ* = 0, where success probability is 1 for any level of effort. In other words, trying to address the consequences of floating duck problem by lowering the difficulty of the world can only result in the world becoming trivially easy.

### 3.2. Individual learning does not fully solve the problem

Another intuitive solution to the floating duck effect is direct individual learning. If individuals get to experience the world themselves, they can get their own unbiased read on the difficulty of the world, counteracting the bias owing to under-reporting of effort. Specifically, we can consider a world where naive individuals first rely on social learning to infer the difficulty of the world, but update their estimates once they have their own experiences. In this world, since individuals decide (and therefore know) both their per activity effort *x* and total effort *X*_*A*_ and observe their successes, they can directly obtain an unbiased estimate of *θ*_*r*_. Thus, we can assume that after just one round of investments, individuals would learn the true difficulty level *θ*_*r*_ without any bias. Note that this analysis is meant to be a ‘best case’ scenario for individual learning; there are many reasons to expect that learning might not yield an unbiased estimate after just one round.

**Figure 5.**
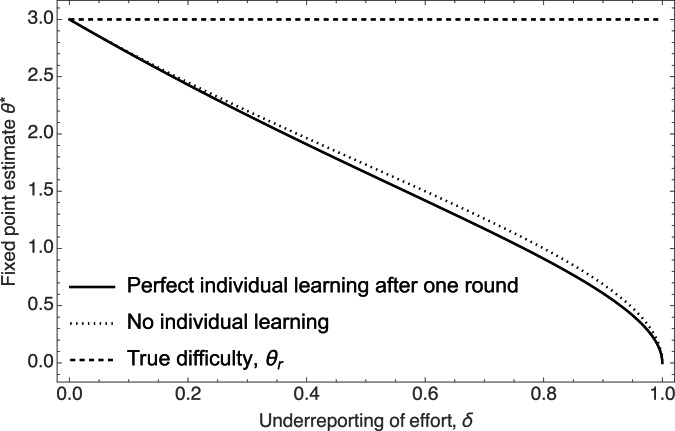
The fixed point estimate for the first round estimate of *θ*, *θ*_1_ in a model with ‘perfect’ individual learning, where individuals after one round can infer the true value of *θ*_*r*_ and invest accordingly. However, the next round individual still observes the average success rate subject to under-reporting of effort. As the figure shows, individual learning by itself does not get rid of the underestimation problem: naive individuals still infer a difficulty of the world lower than the true value (solid curve), and in fact, slightly lower than in the model without any individual learning (dotted curve, same as the top left panel in Figure 4).

Even in this best case scenario for individual learning we run into the trouble when the information gets transmitted through biased observation of effort levels. To see this, we can calculate the average success rate 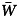 of an individual over two rounds where the individual invests optimally in the first round using socially inferred *θ*_1_ and in the second round using the true *θ*_*r*_:19
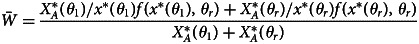
where the numerator is the total number of expected successes over two rounds (first round investing according to an inferred *θ*_1_, second round investing according to the true *θ*_*r*_), and the denominator is the total effort over two rounds. We assume that the next individual observes this average success rate but subject to the same under-reporting of effort *δ* as before (i.e. 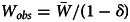 and uses that to infer their initial estimate *θ*_1_. Using the same argument above, we can compute the fixed point for the first round estimate 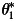. Figure 5 depicts how this first round estimate behaves with *δ*. It shows that while individual learning can solve the problem (by assumption) for the second round investments, under-reporting of effort still will lead individuals to underestimate the difficulty of the world.

## 4. Discussion

In this paper, we use a simple model to elucidate the consequences of the ‘floating duck’ or effortless perfection syndrome, where individuals hide their actual effort and struggles to convey a perception that they achieve their successes with ease. We show that when individuals try to infer the returns to effort, i.e. the difficulty of the world, by social learning from peers, under-reporting of effort leads others to underestimate the true difficulty of the world (i.e. how much effort it takes to succeed), which leads to both increased overall effort but also the spreading out of this effort over too many activities. As a result, we show that individuals might in fact achieve more successes in absolute number, but their success rate, i.e. number of successes per total effort, goes down, because they invest in too many activities. This can explain the twin phenomena of overcommitment and effort–reward imbalances that lead to burnout and anxiety on many education institutions and workplaces.

One alternative hypothesis for overinvestment to the one we model here is that it might arise from an overvaluation of successes, either objectively or owing to biased perceptions. For example, one can easily imagine (and find examples of in the real world) a biased social learning dynamic that leads individuals to overestimate the number of successes they need to get hired for a job (or be admitted to professional schools). This would result in beliefs that overestimate the marginal value of additional successes. In the SI section SI.2, we show that when the return from successes is overestimated individuals also overinvest in aggregate (choose an *X*_*A*_ that is higher than optimal given true value of success). However, they do not overspread the effort (which only depends on the perceived difficulty), so that their probability of success per activity, and therefore their success rate, would not decline. Thus, under the optimal choice assumptions of our model, merely overestimating the value of successes (or overestimating how many successes one needs to get hired) can explain overwork, but cannot explain why effort is spread too thin. The latter requires uncertainty and biased estimates about how effort in each task translates into success in that task under our assumptions. This suggests that other factors, such as uncertainty about overall aptitude of individuals or specific aptitude for particular tasks, might have similar effects. For example, if individuals overestimate their aptitude (which is similar to underestimating difficulty; see SI SI.3), this can lead to both overinvestment and overspreading of effort.

Existing evidence supports some main assumptions and results of our model. Multiple lines of evidence suggest that individuals are indeed not accurately informed about how hard the activities they attempt are, or how their efforts will translate into success. A phenomenon consistently reported across decades is that students tend to overestimate the grades they will receive in classes (Murstein, 1965; Pinquart & Ebeling, 2020), consistent with at least initial underestimation of the difficulty of classes. More recent experimental evidence suggests that individuals change their effort investments when provided with information about the relation between effort and success or rewards, in line with what our model predicts. For example, Ersoy (2023) experimentally induces beliefs of higher vs. lower returns to effort in people who are taking online language learning lessons. Individuals who believed that the returns from lessons are higher (defined as improvement in test scores per lesson) completed more lessons. As this experiment only manipulated beliefs about the returns from a specific activity, this is consistent with our model's prediction that individuals will invest more total effort in an easier world. Likewise, Wright and Arora (2022) find that college students tend to overestimate the grades they will get in a class, and interventions to provide better information about expected grades results in more effort for at least some groups of students. Another recent study by Rury and Carrell (2023) asked students how many hours per week they thought they would have to invest to improve their grade by one letter grade and later told students in the treatment group what this number was in the previous iteration of the same course. Interestingly, they found that most students overestimated how many additional hours they needed to study to improve their grade. Telling them the true number in the previous year therefore lowered how difficult students believed it was to improve their grade (equivalently, increased the expected grade return from an additional hour of study). Again consistent with the model, they found a (short-term) increase in the effort invested in homework, although this effect dissipated over the course of the semester. Overall, these empirical findings support the hypothesis that students often have incorrect estimates of the difficulty of courses or the returns of a given level of effort and react to information about these in a way consistent with our model.

Given its adverse effects, the obvious question is why the floating duck syndrome, specifically the under-reporting of effort, exists in the first place. While our basic model does not directly address this question, it highlights at least two compelling answers. The first answer relies on the observation that the successes achieved are often a means to an end. For example, good grades in college are often regarded as indicators of innate ability and the willingness to put in effort, and prospective employers for example often care about these attributes of a candidate much more than grades or degrees as such. If innate ability and effort are substitutes (as is true in our model and undoubtedly so to some extent in the real world), individuals can manipulate a prospective employer's estimates of innate ability by under-reporting effort. In section SI.3, we present a simple model that illustrates how this phenomenon might work. It shows that under-reporting comes with a tradeoff for a given success level: individuals can appear more capable, but at the same time will appear to have higher effort cost (less willing to put in effort). Yet despite this, the analysis shows that individuals can gain by under-reporting. While that model is only illustrative, it highlights how optimal behavior can lead to downplaying efforts after the fact, setting off the biased learning dynamics and the floating duck syndrome. Our results suggest that we need to design selection and reward schemes and promote cultural change that counteract these incentives to under-report effort. For example, emphasising the willingness to put in effort in the face of difficult tasks relative to having higher innate ability when choosing candidates for jobs and additional educational opportunities would probably reduce the incentive to under-report effort. On the other hand, it can also exacerbate the over-exertion problem. How to navigate this tradeoff remains an open problem.

The second reason for why under-reporting of effort might be prevalent is institutional culture. Notice that our model predicts that the absolute number of successes is increasing the level of under-reporting. This means that individuals at an institution where under-reporting of effort is prevalent would underestimate the difficulty of the world, overinvest, and end up with higher total successes, albeit at the expense of too high individual costs to themselves. Leaders of an institution that measures itself solely by the number of successes of their members would gain by encouraging a culture of under-reporting, as long as the costs borne by members (burnout owing to effort–reward imbalance, negative psychological consequences of excess failure rate) are not reflected in the institution's objective functions. Arguably, this scenario describes many institutions of higher education and many workplaces, at least until relatively recently. A positive development in recent years is that the individual costs of overcommitment and the negative consequences of the floating duck syndrome are becoming more salient for institutions, in part because they start to reflect on the institution itself. It might therefore be expected that these costs, internalised at the institution level, might in the long term weaken or reverse the culture of under-reporting.

We considered some intuitive proposals to counteract the floating duck effect, and found that they do not offer a complete solution. First we can show that the tempting (and oft-taken) short-term fix of lowering the actual difficulty to match the (initially) underestimated levels is doomed to fail in the long term, since it requires constantly lowering difficulty level of tasks until everything becomes trivial. This confirms the intuition that many faculty share that lowering standards is a losing proposition (Arum & Roksa, 2014) but at the same time, our results highlight that simply refusing to lower standards or trying to increase standards does not address the problem, as it simply perpetuates the effort–reward imbalances. Second, letting individuals learn by themselves is also only a partial solution: even if we let individuals gain perfect knowledge of the difficulty of the world from personal experience, as long as this information is transmitted by biased observation of the success rate, social learning dynamics will still lead to initial underestimation of the difficulty. Another tempting solution is to directly reward effort spent on individual activities to avoid overspreading of the effort. Our analyses (not shown) confirm that this can increase the per activity effort for a given difficulty level, but it does not solve the underestimation problem, and therefore at the social learning fixed point individuals will still overinvest and overspread.

Counteracting the under-reporting of effort in social learning dynamics would get to the root of the problem. How this can be accomplished is less clear, however. Cultural change that rewards honest and unbiased accounting of actual effort expended for school tasks would be welcome. For example, revealing what is often called the ‘shadow CV’ (Looser, 2015), which includes not just successfully completed degrees, activities, awards, etc. but also failed activities, e.g. awards, fellowships an individual applied for but did not get, would be helpful. In our model, if all failures are revealed as well as the successes, it allows an unbiased estimate of the difficulty of the world, and counteracts the floating duck effect. However, anecdotal evidence suggests that getting people to reveal all of their failures is a hard sell in contemporary society, for reasons discussed above. At the same time, institutions can invest in accurate accounting of effort that goes into different activities (including extra-curricular activities) and communicating these clearly and in an unbiased way to students to counteract the under-reporting bias.

Our model is clearly highly stylised and does not capture a lot of the complexity of the real world or all mechanisms that can cause overcommitment and burnout for students. This is intentional. Our approach is the time-honoured ‘proof-of-concept’ model approach from evolutionary biology (Servedio et al., 2014), which intends to capture one significant aspect of the real world (in our case, the effects of social learning dynamics based on biased information), while still remaining tractable. Servedio et al. (2014) divides assumptions in this kind of model into three: critical, exploratory and logistical. Our critical assumptions are that individuals are making decisions to maximise their number of successes given that effort is costly, the difficulty of the world is initially unknown to them and they infer it using two pieces of information: the successes of others and their effort level, the latter of which is observed with some downward bias. Our model is intended to show that a seemingly disparate collection of phenomena can be explained with these critical assumptions. These phenomena, which are commonly observed in contemporary college campuses, include individuals both doing too much overall and doing too little for each task, as well as facing feelings of failure even though their absolute number of accomplishments goes up. These results advance a new hypothesis to explain this collection of phenomena based on biases in social learning, rather than individual-level emotional processes.

There are other assumptions that we make that fall into the exploratory and logistical categories. An example of an important exploratory assumption in the simple model is that individuals cannot learn individually. As we show in Section 3.2, adding individual learning from own experience, even under the best case scenario of perfect information gained after just one round, does not fully negate the effects of the biased social learning. Finally, we also made logistical assumptions. For example, we assumed that individuals observe the expected number of successes instead of a draws from a binomial distribution, and that all activities have the same difficulty and reward. Relaxing these would add complexity to the model and might introduce additional wrinkles on top of the basic dynamics discussed here. For example, if activities were heterogeneous in difficulty or reward, individuals would be facing a richer information environment, and depending on how difficulty and reward correlate (and how they are observed), one might imagine additional dynamics like individuals sorting themselves according to their abilities and this might cause additional kinds of biases. Another logistical assumption that we made in our basic model is that the true difficulty of the world, *θ*_*r*_ stays constant at the time scale of the social learning dynamics (except for Section 3.1). If *θ*_*r*_ changes infrequently or slowly, we expect our dynamics to not be affected, as the stability of our social learning dynamics is independent of *θ*_*r*_. However, with large and frequent changes in *θ*_*r*_, the dynamics might not have time to converge to the true value; in this case, the long-run behavior of the population will depend on the process by which the difficulty changes. With very frequeny and large changes, it is possible that social learning might be completely uninformative and individuals might be better off resorting to purely individual learning (Turner et al., 2023). These are interesting future directions to explore.

The main takeaway point from our model is that the widely observed (and seemingly worsening) problems of overcommitment and burnout on college campuses might arise as a consequence individuals learning from their peers in the presence of visibility biases. This has important implications for designing policies; specifically, our model highlights a need to understand peer social learning processes that affect students’ perception of the tradeoffs they face, their time and effort investment decisions, and their measures of success. While social learning has been recognised as a factor for acquiring particular behaviors such as binge drinking (Durkin et al., 2005), we propose that its role extends beyond this. Indeed, recent studies have started looking at social effects on academic success (Stadtfeld et al., 2019), although we are far from a detailed understanding for how students learn from each other and how this affects their decisions about study, work and extracurricular activities. Our model suggests that this is an important area of study to promote more effective educational and career policies, and ultimately, human flourishing.

## Supporting information

Akçay and Ohashi supplementary material Akçay and Ohashi supplementary material

## Data Availability

No data is involved in this study.

## Footnotes

1 This result is true for the stable fixed point  of the social learning dynamics for any *α* > 1. However, equation (17) implies that for *α* > 2, there is a threshold *δ* that is less than 1, above which the fixed point becomes unstable. For *δ*above this threshold, the total number of successes decreases with *δ*.

## Supplementary material

To view supplementary material for this article, please visit https://doi.org/10.1017/ehs.2024.20

## References

[ref1] Adam, M. (2021). *A grounded theory of overcommitment in undergraduate college students*. PhD thesis, Florida Atlantic University.

[ref2] Akçay, E., & Hirshleifer, D. (2021). Social finance as cultural evolution, transmission bias, and market dynamics. Proceedings of the National Academy of Sciences, 118(26), e2015568118. 10.1073/pnas.2015568118 PMC8256012 34172571

[ref3] Arum, R., & Roksa, J. (2014). Let's ask more of our students – and of ourselves. The Chronicle of Higher Education, September 2, A48. https://www.chronicle.com/article/lets-ask-more-of-our-students-and-of-ourselves/

[ref4] Dijkstra, P., Kuyper, H., Van der Werf, G., Buunk, A. P., & van der Zee, Y. G. (2008). Social comparison in the classroom: A review. Review of Educational Research, 78(4), 828–879.

[ref5] Durkin, K. F., Wolfe, T. W., & Clark, G. A. (2005). College students and binge drinking: An evaluation of social learning theory. Sociological Spectrum, 25(3), 255–272.

[ref6] Ersoy, F. (2021). Returns to effort: experimental evidence from an online language platform. Experimental Economics, 24(3), 1047–1073.

[ref7] Ersoy, F. (2023). Effects of perceived productivity on study effort: Evidence from a field experiment. Journal of Economic Behavior & Organization, 207, 376–391.

[ref8] Festinger, L. (1954). A theory of social comparison processes. Human Relations, 7(2), 117–140.

[ref9] Han, B., Hirshleifer, D., & Walden, J. (2023). Visibility bias in the transmission of consumption beliefs and undersaving. The Journal of Finance, 78(3), 1647–1704.

[ref10] Hirshleifer, D., & Plotkin, J. B. (2021). Moonshots, investment booms, and selection bias in the transmission of cultural traits. Proceedings of the National Academy of Sciences, 118(26), e2015571118. 10.1073/pnas.2015571118 PMC8255958 34172573

[ref11] Kendal, R. L., Boogert, N. J., Rendell, L., Laland, K. N., Webster, M., & Jones, P. L. (2018). Social learning strategies: Bridge-building between fields. Trends in Cognitive Sciences, 22(7), 651–665. 29759889 10.1016/j.tics.2018.04.003

[ref12] Lavy, V. (2023). The effect of multitasking on educational outcomes and academic dishonesty. Technical report, National Bureau of Economic Research.

[ref13] Looser, D. (2015). Me and my shadow CV: What would it look like with not just the successes of my professional life but also the many rejections? The Chronicle of Higher Education, 62(10), A26–A27.

[ref14] McClelland, L. E., & Case, K. F. (2023). Is class worth their time? College student perspectives on class structure and attendance. Studies in Educational Evaluation, 78, 101281.

[ref15] Mistler, B. J., Reetz, D. R., Krylowicz, B., & Barr, V. (2012). The association for university and college counseling center directors annual survey. Retrieved from Association for University and College Counseling Center Directors.

[ref16] Murstein, B. I. (1965). The relationship of grade expectations and grades believed to be deserved to actual grades received. The Journal of Experimental Education, 33(4), 357–362.

[ref17] Nordentoft, M., Rod, N. H., Bonde, J. P., Bjorner, J. B., Madsen, I. E., Pedersen, L. R., … Rugulies, R. (2020). Effort–reward imbalance at work and risk of type 2 diabetes in a national sample of 50,552 workers in denmark: A prospective study linking survey and register data. Journal of psychosomatic research, 128, 109867. 31715495 10.1016/j.jpsychores.2019.109867

[ref18] Pinquart, M., & Ebeling, M. (2020). Students’ expected and actual academic achievement – a meta-analysis. International Journal of Educational Research, 100, 101524.

[ref19] Porru, F., Robroek, S. J., Bültmann, U., Portoghese, I., Campagna, M., & Burdorf, A. (2021). Mental health among university students: The associations of effort–reward imbalance and overcommitment with psychological distress. Journal of Affective Disorders, 282, 953–961. 33601740 10.1016/j.jad.2020.12.183

[ref20] Rury, D., & Carrell, S. E. (2023). Knowing what it takes: The effect of information about returns to studying on study effort and achievement. Economics of Education Review, 94, 102400.

[ref21] Scelfo, J. (2015). Suicide on campus and the pressure of perfection. *New York Times,* July 27. https://www.nytimes.com/2015/08/02/education/edlife/stress-socialmedia-and-suicide-on-campus.html

[ref22] Servedio, M. R., Brandvain, Y., Dhole, S., Fitzpatrick, C. L., Goldberg, E. E., Stern, C. A., … Yeh, D. J. (2014). Not just a theory – the utility of mathematical models in evolutionary biology. PLoS Biology, 12(12), e1002017. 25489940 10.1371/journal.pbio.1002017 PMC4260780

[ref23] Siegrist, J. (1996). Adverse health effects of high-effort/low-reward conditions. Journal of Occupational Health Psychology, 1(1), 27. 9547031 10.1037//1076-8998.1.1.27

[ref24] Stadtfeld, C., Vörös, A., Elmer, T., Boda, Z., & Raabe, I. J. (2019). Integration in emerging social networks explains academic failure and success. Proceedings of the National Academy of Sciences, 116(3), 792–797. 10.1073/pnas.1811388115 PMC6338824 30584099

[ref25] Stanford University Student Affairs (2022). In focus: Don't be a duck! How to resist the Stanford Duck Syndrome. https://studentaffairs.stanford.edu/the-flourish/flourish-october-2022/focus-dont-be-duck-how-resist-stanford-duck-syndrome (accessed 31 July 2023).

[ref26] Stinebrickner, R., & Stinebrickner, T. R. (2008). The causal effect of studying on academic performance. The BE Journal of Economic Analysis & Policy, 8(1), 14.

[ref27] Strogatz, S. (1994). Nonlinear dynamics and chaos: With applications to physics, biology, chemistry, and engineering. Studies in Nonlinearity. Addison-Wesley.

[ref28] Tullock, G. (1980). Efficient rent seeking. In Buchanan, J. M., Tollison, R. D., & Tullock, G., (Eds.), Toward a theory of the rent-seeking society (pp. 97–112). Texas A&M University.

[ref29] Turner, M. A., Moya, C., Smaldino, P. E., & Jones, J. H. (2023). The form of uncertainty affects selection for social learning. Evolutionary Human Sciences, 5, e20. 37587949 10.1017/ehs.2023.11 PMC10426062

[ref30] Verduyn, P., Gugushvili, N., Massar, K., Täht, K., & Kross, E. (2020). Social comparison on social networking sites. Current Opinion in Psychology, 36, 32–37. 32387840 10.1016/j.copsyc.2020.04.002

[ref31] Wege, N., Li, J., Muth, T., Angerer, P., & Siegrist, J. (2017). Student eri: Psychometric properties of a new brief measure of effort–reward imbalance among university students. Journal of Psychosomatic Research, 94, 64–67. 28183404 10.1016/j.jpsychores.2017.01.008

[ref32] Wright, N. A., & Arora, P. (2022). A for effort: Incomplete information and college students’ academic performance. Economics of Education Review, 88, 102238.

