# Supplementary material for: The floating duck syndrome: biased social learning leads to effort–reward imbalances
Source: Evol Hum Sci. 2024 Apr 29;6:e30. doi: 10.1017/ehs.2024.20 (PMC11362996; doi:10.1017/ehs.2024.20)
Supplement: Akçay and Ohashi supplementary material [file S2513843X24000203sup001.pdf]

Supplementary Information

for

The floating duck syndrome: biased social learning leads to effort-reward imbalances

Erol Akçay* and Ryotaro Ohashi

*: corresponding author: eakcay@sas.upenn.edu

SI.1 Change in the optimal efforts with θ under perfect information

In this section, we show that the comparative statics of the basic model hold more generally than the specific functional form of the Tullock contest function presented in the main text. Specifically, show that optimal total investment is decreasing while investment per activity is increasing with the difficulty of the world.

To do that, consider the first order conditions (6) and (7), where the optimal effort levels $X_A^*(\theta)$ and $x^*(\theta)$ are functions of θ, the difficulty of the world. We can take the total derivative of equations (6) and (7) with respect to θ, which gives:

$$\frac{1}{x^*}\left[\frac{f\left(x^*,\theta\right)}{x^*}-\frac{\partial f}{\partial x}\right]\frac{dx^*}{d\theta}+\frac{\partial X_A}{\partial\theta}\frac{\partial^2 c}{\partial X_A^2}=\frac{1}{x^*}\frac{\partial f}{\partial\theta} \tag{SI.1}$$

$$\left(\frac{\partial^2 f}{\partial x^2}+\frac{\partial^2 f}{\partial x\partial\theta}\right)\frac{dx^*}{d\theta}+\frac{1}{{x^*}^2}\left[\frac{f\left(x^*,\theta\right)}{x^*}-\frac{\partial f}{\partial x}\right]\frac{dx^*}{d\theta}=\frac{1}{x^*}\frac{\partial f}{\partial\theta}\,, \tag{SI.2}$$

where all the derivatives are evaluted at $x = x^*(\theta)$ and $X_A = X_A^*(\theta)$. Observe that in the left hand side of both equations, the square brackets are exactly the first order condition (7), such that the brackets vanish. Then we can solve for $\frac{\partial X_A}{\partial\theta}$ and $\frac{dx^*}{d\theta}$ to get:

$$\frac{dx^*}{d\theta}=\left(\frac{1}{x^*}\frac{\partial f}{\partial\theta}-\frac{\partial^2 f}{\partial x\partial\theta}\right)\Big/\frac{\partial^2 f}{\partial x^2} \tag{SI.3}$$

$$\frac{\partial X_A^*}{\partial\theta}=\frac{1}{x^*}\frac{\partial f}{\partial\theta}\Big/\frac{\partial^2 c}{\partial X_A^2}\,, \tag{SI.4}$$

where all derivatives are evaluated at $x^*(\theta)$ and $X_A^*(\theta.k)$. Provided that $\frac{\partial^2 f}{\partial x\partial\theta}\geq 0$[2], these equations imply that the optimal total effort $X_A^*(\theta,k)$ is decreasing in θ, while the optimal effort per activity $x^*(\theta)$ is increasing. In other words, if the world is more difficult, individuals will invest more into a given activity but invest less effort overall, which will result in fewer activites attempted. Likewise, we have for the expected number of successes at given optimal efforts, $s^*(\theta,k)=s(X^*(\theta,k),x^*(\theta),\theta)$:

$$\frac{ds^*}{d\theta}=\frac{\partial X_A^*}{\partial\theta}\frac{1}{x^*}f(x^*(\theta),\theta)+\frac{X_A^*(\theta)}{x^*(\theta)}\frac{\partial f}{\partial\theta}<0\,, \tag{SI.5}$$

[2] This condition is sufficient but not necessary. It will be generally satisfied for sigmoidal functions at the optimal effort levels, which will be to the right of the inflection point.

where the last inequality follows from the fact that both $\frac{\partial X_A^*}{\partial \theta} < 0$ and $\frac{\partial f}{\partial \theta} < 0$. This, the expected number of successes goes down with increasing difficulty of the world, as is intuitive.

SI.2 Overvaluation of successes does not explain overspreading of effort

In this section, we show that a reasonable alternative hypothesis for overinvestment, the over valuation of successes, does not reproduce the second component of the effort-reward imbalance: the reduction in success rate per effort. To show this, we assume that the individuals know the true difficulty of the world θ_r, but believe that the return from their successes is given by a function $g(s, \rho)$, where ρ is a parameter that determines the marginal rewards from success. For example, the function $g(\cdot)$ can be of the same logistic type as the success function $f(\cdot)$ from each activity, and denote the probability of getting a good job, or getting into graduate school. Then, ρ could be analogous to θ, denoting the difficulty of achieving that goal. Mathematically, we represent these assumptions as $\frac{\partial g}{\partial s} > 0$ and $\frac{\partial^2 g}{\partial s \partial \rho} > 0$. The first inequality means more successes leads to more rewards, while the second means that increasing ρ increases the marginal returns from more successes (although the independence of the success per activity result only require the first inequality).

The first order conditions for optimal investments are then:

$$\frac{\partial g}{\partial s}\frac{\partial s}{\partial X_A} - \frac{\partial c}{\partial X_A} = 0 \tag{SI.6}$$

$$\frac{\partial g}{\partial s}\frac{\partial s}{\partial x} = 0\,. \tag{SI.7}$$

Note that these equations differ from our baseline case (equations (6) and (7)) only in the inclusion of the $\frac{\partial g}{\partial s}$, which modulates the marginal value of the successes. Further, our first order condition (SI.7) for the investment per activity, x, can be simplified to be exactly identical to (7), since we assume $\frac{\partial g}{\partial s} > 0$. Given that the function f is independent of ρ, it follows that the optimal investment per activity is also independent of ρ.

On the other hand, one can take the total derivative of equation (SI.6) with respect to ρ (noting again that f is independent of ρ and X_A) to find:

$$\frac{\partial X_A^*}{\partial \rho} = \frac{x^* f(x^*, \theta)\frac{\partial^2 g}{\partial s \partial \rho}}{x^{*2}\frac{\partial^2 c}{\partial X_A} - f(x^*, \theta)^2 \frac{\partial^2 g}{\partial s^2}} > 0\,, \tag{SI.8}$$

where the inequality at the end follows from the assumption that $\frac{\partial^2 g}{\partial s \partial \rho} > 0$ and the second order condition $\frac{\partial^2}{\partial X_A^2}\left(g(s) - c(X_A, k)\right) < 0$ which makes the denominator positive. Thus, total effort will increase with overvaluation of success (higher ρ) but effort per activity, and therefore the success rate (given by $\frac{f(x^*,\theta)}{x^*}$) will remain unchanged with ρ. This proves that if overinvestment of effort flows purely from overvaluation of successes, it does not generate the kind of effort-reward

imbalance that underestimating the difficulty of the world does.

SI.3 The incentive to underreport effort

In this section, we consider a slight elaboration of the model in the main text to illustrate why agents can have an incentive to underreport their actual effort levels. Specifically, we present a simple model of the idea proposed in the Discussion that agents might differ in their intrinsic ability, and might be tempted to appear more able than they are by claiming to have achieved their successes with lower effort than they actually spent. We will show that they can use such underreporting to make prospective employers (or academic advisors) have higher expectations of success from them. If a prospective employer bases their decisions on this expectation of successes, individuals would be incentivized to underreport their efforts.

As our starting point, we take our basic model with perfect information, i.e., assume individuals know the true difficulty of the world θ_r. To this model, we add individual variation in ability, which –like the cost parameter k– is privately known. Specifically, we endow each individual with ability η, such that the success function for that individual is given by:

$$f(x, \theta_r/\eta) = \frac{x^a}{x^a + \left(\frac{\theta}{\eta}\right)^a} . \tag{SI.9}$$

Thus, for a given θ_r, a higher value of η will make the world appear easier to the individual, and the same effort will have a higher probability of success for higher η. We then compute the optimal allocation of effort using the first order conditions (6) and (7), with the only difference being the replacement of θ by θ/η.

Now we assume that a prospective employer knows the true difficulty of the world the candidate faced θ_r (either from personal experience or experience with other candidates), as well as the difficulty of the job they are offering, which we will call θ_j. But the prospective employer does not know η and has to infer this as well as the cost parameter k of the candidate to be able to form an expectation of the candidate's success probability. This problem is mathematically very similar to the one solves in the main text (Section "Inferring the difficulty of the world in a heterogenous population") and we can show that with the same information as in that section (number of observed successes s_{obs} and effort $X_{A,obs}$), the employer can infer both η and k, under the assumption that the candidate invested optimally and reported turthfully. These estimates (using the Tullock contest function above and quadratic costs as in the main text) are:

$$\eta_{est} = (a-1)^{\frac{1}{a}-1} a\theta \frac{s_{obs}}{X_{A,obs}} \tag{SI.10}$$

$$k_{est} = \frac{s_{obs}}{2X_{A,obs}^2} . \tag{SI.11}$$

With underreporting of effort, $X_{A,obs} = (1-\delta)X_{A,true}$, so these expressions confirm that by under-

reporting effort they spent to achieve a given number of successes, a candidate can appear to be more capable (higher η). However, this comes with a trade-off, the candidate also appears that effort is more costly to them (higher k); in a way, the candidate appears "lazier."

What happens next depends on how the employer is setting the job parameters, and how the employer is compensating the candidate, which determine what the objective function of the employer and the candidate are. Below we consider two simple models.

Model 1: compensation for effort, constant difficulty job

The first model is one where the employer compensates the candidate for effort at a constant rate (e.g., hourly payments), and that the the candidate does not care about the success of the job in itself. The candidate will instead put in the effort that maximizes their earnings. If the employer pays σ per unit effort, the candidate's objective function (as estimated by the employer) becomes:

$$\sigma x_j - k_{est} x_j^2 \,, \tag{SI.12}$$

and the expected effort from the candidate would be $x_j^* = \sigma / 2k_{est}$. The employer can then solve for the optimal compensation rate σ^* that maximizes the employer's objective function (the probability of success given expected efforts of the candidate minus the wages paid at that effort level):

$$f(x_j^*, \theta_j/\eta_{est}) - \sigma x_j^* \,. \tag{SI.13}$$

Here, both x_j^* and η_{est} will depend on δ, and therefore σ^* will also depend on δ. Figure SI.1 shows that the optimal compensation for the employer is increasing in the underreporting, δ. Intuitively, this is because with underreporting, the candidate both appears less willing to work (so will require higher rate of compensation), but at the same time more capable, which means that the employer is willing to pay a higher compensation. Given that the candidate's true cost function is fixed, the candidate will always prefer a higher wage (which will make the candidate work harder, but also earn more). This shows how a candidate might be incentivized to underreport their effort given their realized successes.

Model 2: compensation for success, varying difficulty job

An alternative compensation scheme is where the employer pays only for successes, but assigns the candidate to a job based on their abilities where more able candidates get assigned to more difficult but more rewarding jobs. Specifically we may assume that the employer assigns the candidate to a job of difficulty η_{est} so that the employer expects the candidate to always face a subjective difficulty of 1. Further, assume that each job has reward equal to its difficulty and the employer compensates the candidate with a fraction σ of the reward in the event of success (and zero in the event of failure). Then, the objective function of the candidate (as seen by the employer)

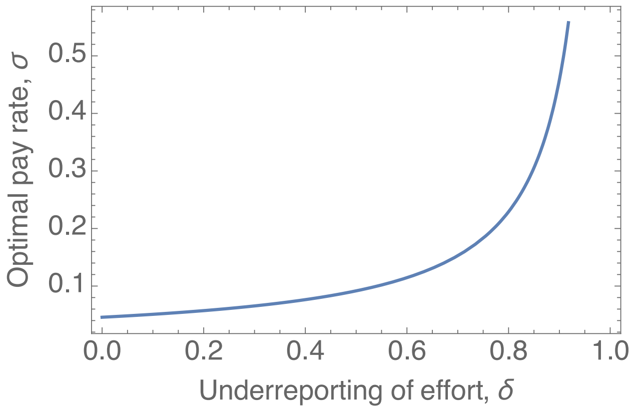

Figure SI.1. The change in optimal compensation for the employer with the underreporting of effort by the candidate. Here, the difficulty of the initial world and the job are the same $\theta_r = \theta_j = 3$ with $a = 2$, and the candidate has true ability $\eta = 1$ and cost parameter $k = 1/200$. We assumed that the candidate knows η_r and has invested optimally in the first round, and used the expected success and optimal effort rates to calculate η_{est} and k_{est} as a function of underreporting δ. We then fed these into the objective function of the employer and calculates σ^* as a function of δ, as described in the text.

becomes

$$\sigma \eta_{est} f(x_j, 1) - k_{est} x^2 \,, \tag{SI.14}$$

while the employer's own objective function is

$$(1 - \sigma)\eta_{est} f(x_j, 1) \,. \tag{SI.15}$$

We can again compute the optimal σ^* for the employer (maximizing expression (SI.15)) under the assumption that the employer expects the candidate to behave optimally given estimated ability and cost parameters (which, again, are functions of δ). In this setting, we have to also consider the candidate's actual optimal behavior, which will be different than what the employer expects. This is because an underreporting candidate will be assigned to a job with diffuculty higher than their true ability, and will also have a lower cost function. Therefore they might experience a higher failure rate. Going through these calculations for $a = 2$, we can show that at least under some parameters, candidates indeed can gain by underreporting their effort, being seen as higher ability, and being assigned to more difficult jobs with higher rewards (Figure SI.2). This happens because the two effects of underreporting go in the same direction: the candidate both gets assigned a potentially more rewarding job, and gets compensated at a higher rate for success because the employer thinks the candidate is harder to motivate. However, the expected (true) success rate of the candidate is lower with underreporting, contributing again to the effort-reward imbalance.

We must point out that the simple models in this section are meant to be illustrative of the incentives, and not a comprehensive model for how employers might compensate candidates, and other factors might counteract or complicate the incentive to underreport. For example, in

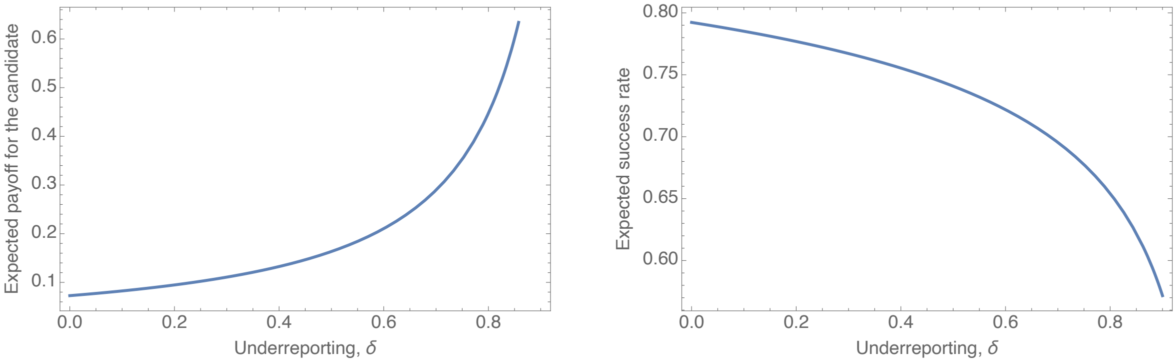

Figure SI.2. The expected payoff of the candidate (left panel) and the expected success rate (right panel) as a function of the underreporting of effort to a prospective employer. Here again, the difficulty of the world prior to employment $\theta_r = 3$, the candidate has true ability level $\eta = 1$ and cost parameter $k = 1/200$, and is assumed to have made optimal investment decisions with accurate knowledge of the difficulty level. The success function has shape parameter $a = 2$.

the compensating effort model above, a candidate wins by underreporting when negotiating over salary, but one can show that at the optimal compensation level, the expected success (from the employer's point of view) at the job stays constant in δ. That implies that if the employer faces a choice between multiple candidates with the same true (but unobservable to the employer) ability and cost parameters, the employer should prefer the one with less underreporting, as they would get the same success at a lower cost. This would counteract some of the incentives to underreport, but given that the employer will not generally know the true η and k and will face a heterogenous pool of applicants with different amount of successes, the incentive to underreport will remain.
